# Green Infrastructure Designed through Nature-Based Solutions for Sustainable Urban Development

**DOI:** 10.3390/ijerph20021102

**Published:** 2023-01-08

**Authors:** Snežana Štrbac, Milica Kašanin-Grubin, Lato Pezo, Nataša Stojić, Biljana Lončar, Ljiljana Ćurčić, Mira Pucarević

**Affiliations:** 1 Institute of Chemistry, Technology and Metallurgy, University of Belgrade, 11000 Belgrade, Serbia; 2 Institute of General and Physical Chemistry, University of Belgrade, 11000 Belgrade, Serbia; 3 Faculty of Environmental Protection, Educons University, 21208 Sremska Kamenica, Serbia; 4 Faculty of Technology Novi Sad, University of Novi Sad, 21000 Novi Sad, Serbia

**Keywords:** nature-based solutions, urban green infrastructure, ecological index, energy plantations, sustainable drainage system

## Abstract

With the goal of enhancing the quality of the environment, urban green infrastructure (UGI) is an essential element in sustainable cities, and nature-based solutions (NBS) are being carried out as new infrastructure solutions that increase the resilience of cities. In this research, the method of theoretical analysis and the content analysis as the basic fact-gathering technique was applied to answer to following questions: What are the hindrances and bottlenecks in implementing NBS? Are the current decision-making mechanisms helping NBS get in route to shape cities? Is there any binding policy in practice that promotes NBS? In Belgrade is planned Type 3 of the degree of intervention/level and engineering type—Creation and new ecosystem management in the classifications of intensive urban green space management; urban planning strategies; urban water management; ecological restoration of degraded terrestrial ecosystems; and restoration and creation of semi-natural water bodies and hydrographic networks. In the future, it is essential to implement policies and incentives on national, regional, and local scales that help encourage the usage of NBS in the development of urban infrastructure.

## 1. Introduction

In cities that consider new approaches to the development of infrastructure, nature-based solutions (NBS) have become a valid alternative to traditional approaches [1]. NBS are being carried out as new infrastructure solutions aimed at addressing challenges related to climate change, ecosystem resilience, human well-being, and health. They are defined as activities motivated by, supported by, or replicated from nature [2,3] that deploy different natural attributes and processes in a resource-efficient and sustainable manner; are adjusted to local systems into diverse spatial scales redefining the role of nature in urban, rural, and natural environments; and contribute multiple benefits and support sustainable development when faced with social, environmental, and economic demands. NBS is a valuable tool for achieving sustainability objectives such as sustainable urbanization and reviving degraded ecosystems. Urban renaturing may be a valuable strategy to radically transform the landscape. Renaturing is based on a procedure of spatial modification resulting from the expansion of nature (flora, fauna, water, soil, microbes, fungi, habitats) and the restoration of ecological functioning in human environments. All of this is achieved by integrating established ecosystem-based approaches (e.g., ecosystem service protection, biodiversity conservation, and green infrastructure). In this way, the NBS is used for a holistic approach to economic, social, and environmental issues, as well as increasing cities’ resistance to future threats. Three types of NBS share the ambition of enhancing the delivery of a range of ecosystem benefits: resolutions with no or minimal intervention in ecosystems; interventions in managed ecosystems and landscapes aiming for sustainability and multifunctionality; and regular design or profound modification of ecosystems to create green or blue infrastructure [4]. 

For the challenges posed by environmental degradation and climate change, urban green infrastructure (UGI) through NBS has the potential to bring multiple benefits to urban areas. UGI as a method of providing economic, ecological, and social benefits through natural solutions can make a significant contribution to the implementation of all policies whose goals can be achieved through NBS. UGI is a network of human-managed and natural ecosystems that improve an ecosystem’s health and resilience, contribute to biodiversity, and help human populations by supporting and enhancing an ecosystem’s benefits [5,6]. Additionally, UGI can be described as landscape features that can deliver environmental, social, and economic advantages [7]. The main task of UGI strategies is to help societies understand the value of the benefits that nature provides, and also in response to organize the investments necessary to maintain and improve those benefits. In cities, UGI can help deliver a large number of environmental benefits specifically reflected in ecosystem services such as regulating and provisioning services [8,9]. These especially include clean air and improved water quality. UGI has a positive effect by absorbing large amounts of carbon dioxide, contributing to climate change mitigation via carbon sequestration, reducing other pollutants from the air, affecting the temperature and humidity, improving the quality of groundwater, providing a reliable supply of clean drinking water and natural circulation of atmospheric water, providing a place for life, reproduction, and food for species of flora and fauna, preventing soil erosion, reducing noise and wind, and supporting human health by creating suitable places for people to live a healthy life [10,11]. Thanks to the physiological processes of plants (absorption, evaporation, transpiration, photosynthesis, etc.) UGI helps to mitigate the effects of “local heat islands” as “living technology” [7]. In this way, UGI represents the “climate infrastructure” of cities [12]. Social benefits are reflected in the fact that UGI contributes to people’s mental and physical well-being [13]. UGI can contribute to improving mental health by strengthening communities and combating social isolation and exclusion. As a space of natural, cultural, and aesthetic values, UGI constitutes places of meeting, contact, communication, education, recreation, and enjoyment for the inhabitants, having a positive effect on people’s psychophysical health and affirming the social dimension of the city [12]. Cultural heritage could entail conventional human-influenced topography, inventive NBS, and beneficial natural areas. For instance, urban national parks often contain various cultural, historical, and natural values, and they propose the significant possibility of incorporating NBS, where nature can be integrated into additional forms [12]. Thanks to UGI, an urban–rural matrix can be designed that helps urban areas become more attractive places to live. Finally, UGI solutions are key to increasing disaster resilience (e.g., fires and floods). Innovative risk management enables adaptation to the risks associated with climate change, and UGI represents an opportunity for such investment. All of these highlight the importance of systematically including and considering UGI in planning and decision-making processes. Urban forests, public parks, city trees, community gardens, green barriers, green corridors, urban wetlands, and urban protected areas (UPA), etc. represent UGI [14,15]. Sustainable drainage systems (SuDS), energy plantations of fast-growing deciduous tree species, systems for wastewater treatment, and revitalization of polluted water through plants also can be considered elements of UGI, and, at the same time, they are crucial elements for mitigating the consequences of climate change.

UGI and NBS are increasingly recognized for their potential to contribute to a range of urban challenges and policy objectives in many cities. In the countries of Central-Eastern Europe, such as Romania and Croatia, half of the population lives in urban areas, while in Hungary over 70% of the population lives in cities. In Belgrade (Serbia), existing public green areas and forests are good of general interest, which is why it is necessary to preserve, improve, and plan them as unique systems of green areas. The spatial realization should result in the establishment of a system of different interconnected types of green areas. The main objective of this paper was to present a case study from an urban environment that deals with the local future-planned city regeneration process with the function of changing the physical organization, improving biodiversity, ecosystem health, and resilience, and mitigating climate change, all through the prism of the implementation of NBS. This study focused on answering the following questions: What are the hindrances and bottlenecks in implementing NBS? Are the current decision-making mechanisms helping NBS get in route to shape cities? Is there any binding policy in practice that promotes NBS? The answers to these questions can contribute to effective changes that must be rapidly developed in cities in order to raise awareness of the importance of UGI and NBS in the mitigation of future environmental threats. 

## 2. Methods

To obtain an answer of how UGI, through NBS, is mainstreamed in the future urban infrastructure development practice of the city of Belgrade and to what extent Belgrade’s current city policy framework supports their implementation, in this research, the method of theoretical analysis was applied. The content analysis and case study were applied as the basic fact-gathering technique [16]. Content analysis is a special type of quantitative empirical research aimed at determining the presence of certain ideas, ideological tendencies, their holders and opponents, and, most often, the prevailing discourse in some areas of social life. The content analysis includes, of course, qualitative research elements, considering that any content analysis must start from a clear identification of categories [16]. A case study is an empirical investigation that investigates a contemporary phenomenon within its real-life context, especially when the boundaries between phenomena and context are not clearly discernible. This allows the forecasting of short-term and long-term conditions, behavior, and events related to the context of the given case and/or similar cases [16]. 

### 2.1. Theoretical Framework

In order to comprehend the future-planned local regeneration of the city in terms of changes in physical organization, improvement of biodiversity, ecosystem health and resilience, and mitigation of climate change, all through the prism of NBS implementation, the authors set out to review the landscape of policy documents in Belgrade, Serbia. The data were drawn from policy documents such as the Plan of general regulation of the green areas of the city of Belgrade (2019), the Climate change adaptation action plan with vulnerability assessment (2015), the Environmental protection program of the city of Belgrade (2015), and Strategy of development of the city of Belgrade: strategic goals, priorities, and measures for sustainable development until 2021 (2018).

The reason for the development of the Plan of general regulation of the green areas of the city of Belgrade (2019) is the strategic commitment of Belgrade that the city should be planned based on the principles of sustainability. The concept of development defines that the realization of the system of green areas implies a change of their status from a subordinate to the primary city infrastructure; in addition to the norms that control the planning system, it is necessary to develop city “green regulations”; the conception and organization of the green area system will be realized through further planning elaborations, detailed studies, recording and valorization of existing and potential green areas, and the state of the environment [12]. The Development of an Action plan for adaptation to climate change with vulnerability assessment (2015) represents only one, but nonetheless very important step in a series of measures to be implemented to establish a system of adaptation to address climate change, which will have a positive impact on the whole range of aspects required to raise living standards (environmental protection and human health, improving environmental space, preservation of material values, economic aspects, etc.) [17]. The purpose of the Environmental protection program of the city of Belgrade (2015) is to obtain a strategic document in the field of protection, improvement, and management of environmental protection, which determines the situation in this area, identifies trends, defines goals, and ensures the consistency of management and environmental policy with other sectoral policies. The program is focused on solving the problems that put the greatest pressure on the environment and represent the first step in the long-term management of the relationship between society and nature [18]. The main goal of Belgrade’s city development strategy for the next five years is to enhance citizens’ quality of life and improve economic performance with continuous investment, improvement of identity, affirmation of public space, riverbanks, and coastal zones of the city [19].

### 2.2. Case Study—City of the Belgrade

**Figure 1:**
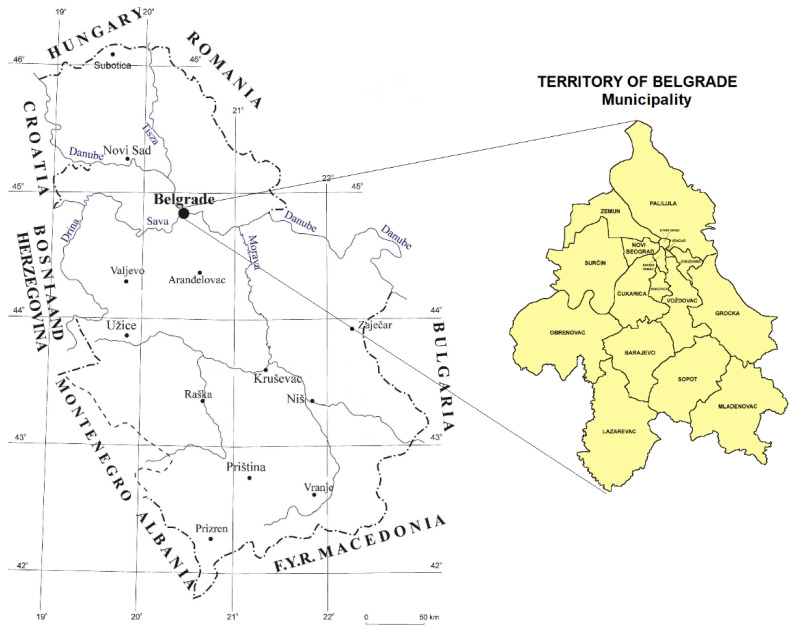
The territory of Belgrade (https://www.zdravlje.org.rs/ekoatlas/volbe/02ev.gif, accessed on 11 August 2020).

The city of Belgrade was chosen as a case study in this research. The city of Belgrade, the capital of the Republic of Serbia, is the center of state administration. The functional area of Belgrade covers 7.4% of Serbian territory (5758 km), and a quarter of the population (25.4%) lives there [20]. The area of the city is administratively divided into 16 city municipalities (Figure 1). The great economic potential of the city makes it an excellent geostrategic position, on two European Rivers (the Sava and Danube), where two of the ten European corridors intersect (VII and X with branch XI) [18]. Belgrade’s total population is 1,659,440 [21].

In terms of geomorphology, the area of Belgrade is very complex. The following basic geological–geomorphological categories of terrain are represented on the territory of the city: low-lying terrains of fluvial genetic type; aeolian and aeolian–aquatic plateaus of southern Srem; neogene hilly terrain; hilly and hilly-mountainous terrains, and terrains formed by anthropogenic activity. The lowest parts of the terrain are the alluvial plains of the Sava and the Danube Rivers, while in Belgrade hills, the peaks of the Avala and Kosmaja mountains stand out. 

Belgrade’s vegetation is viewed through functional–ecological units, i.e., biomes. Three types of biomes can be distinguished. In the north, there is a steppe biome and forest steppe. Along the watercourses exists the biome of the southern European deciduous forests of the floodplain and lowland type. In the south, there is a biome of sub-Mediterranean forests with malt and oak [22].

Belgrade is characterized by a moderate continental climate, with pronounced differences between the average annual temperatures in summer and winter. Summers are warm and temperatures above 30 °C usually last an average of 31 days a year, and temperatures above 25 °C last an average of 95 days. Winters are cold and snowy with an average of 21 days a year below 0 °C. Therefore, Belgrade significantly contributes to the effects that cause climate change but also suffers from the consequences of climate change. Due to the high level of urbanization, the growing share of built-up areas, greenhouse gas emissions, as well as the growing area of impermeable substrates, climate change is becoming more visible to ordinary citizens and affecting the quality of life [22].

Belgrade’s natural resources include agricultural and forest land, significant mineral resources, and water resources. There is 214,429 ha of agricultural land on the territory of the city, which makes up 66.45% of the total territory. The function of forests in Belgrade is not only generally useful in terms of improving the environment, preserving biodiversity, contributing to improving air quality and mitigating the effects of “greenhouse” from the effects of climate change, protecting land from erosion, landslides, and floods, and creating favorable conditions for human health, but they also constitute one the most important sources of economic activities. The water areas of the city of Belgrade include parts of the Danube River basin, parts of the Sava sub-basin, and parts of the watercourse sub-basins located within the administrative boundaries of Belgrade [23]. The surface waters of Belgrade appear in the form of large and small watercourses that intersect or frame the territory of the city, a large number of small watercourses that occur on its territory, as well as several lakes and other surface reservoirs [18]. 

The territory of the city of Belgrade is characterized by a relatively diverse, and in economic terms, very significant mineral wealth. The Kolubara basin lignite deposit is of great importance [22]. 

On the territory of Belgrade, 46 areas are protected by law, of which 36 sites are protected as natural monuments, 3 sites are areas of exceptional features, 5 sites are protected as geological nature monuments, 2 sites are nature reserves, and 6 sites are protected environments of the immediate cultural property. The protected areas occupy 1.76% of the total area of the city [18].

#### Ecological Characteristics of Belgrade

**Figure 2:**
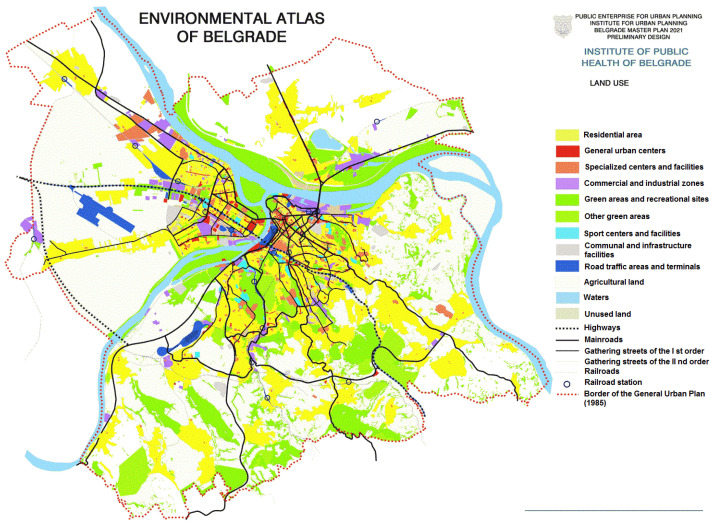
Land use (https://www.zdravlje.org.rs/ekoatlas/volbe/03ev.gif, accessed on 23 August 2022).

The state of the environment of Belgrade is determined by its natural conditions, urban and physical structure, economic activities, traffic, and socio-economic processes that take place in the city and its surroundings. Zones with different qualities of the environment can be distinguished in Belgrade: areas of the preserved environment under the protection regime (protected areas); areas of preserved environment without a formal protection regime; urban spaces (narrower city core); peri-urban belts; rural areas; and areas of degraded environment (Figure 2). Thermal power plants and the accompanying infrastructure, industrial plants and brownfield sites, oil refineries and storage depots of petroleum products, illegal landfills, roads, and marshaling yards are the dominant pollutants causing a negative impact on the environment (Figure 2). The lack of adequate communal infrastructure directly causes pollution of the land and watercourses in all localities.

**Figure 3:**
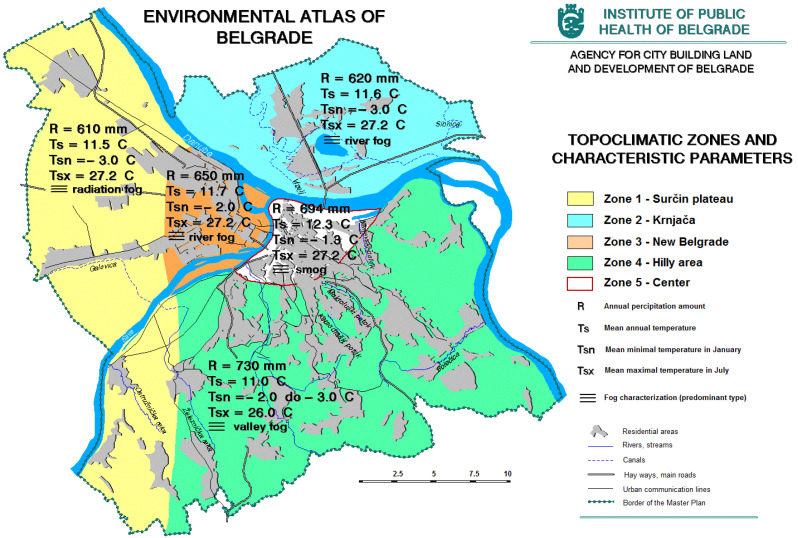
Topoclimatic zones of Belgrade (https://www.zdravlje.org.rs/ekoatlas/volbe/17ev.gif, accessed on 23 August 2022).

**Figure 4:**
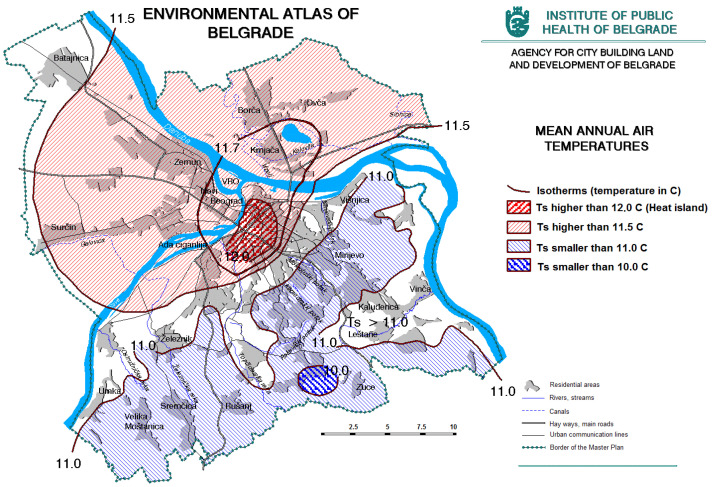
Mean annual air temperatures (https://www.zdravlje.org.rs/ekoatlas/volbe/16ev.gif, accessed on 23 August 2022).

Climate parameters (temperature, precipitation regime, sunshine, local wind roses, etc.) significantly affect the quality of the environment in the city. Changes in two important climatic parameters, temperature, and precipitation, certainly have the most significant impact on the quality of life of people in the urban environment. For Belgrade is an evident increase in average monthly and average annual temperature, which is, among other things, a consequence of urbanization (Figure 3 and Figure 4). Changes in precipitation regimes do not have such a clear trend (Figure 3). The ratio of the maximum and minimum annual amount of precipitation indicates that large urban areas have the effect of increasing extremes—dry years in the wider area are even drier in cities, and those with increased precipitation tend to have extreme values [12]. 

**Figure 5:**
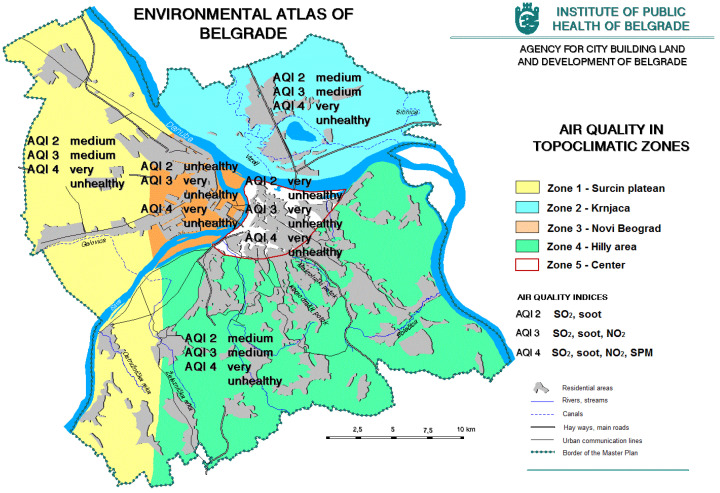
The air quality of Belgrade (https://www.zdravlje.org.rs/ekoatlas/volbe/36ev.gif, accessed on 23 August 2022).

The main factors threatening the air quality in Belgrade are stationary and mobile sources. In terms of air pollution, the most endangered parts of the city are the central zone of the old town, as well as roads with a higher load (Figure 5). The entire population is exposed to polluted air, and vulnerable groups, children, the sick, and the elderly are particularly at risk [12].

**Figure 6:**
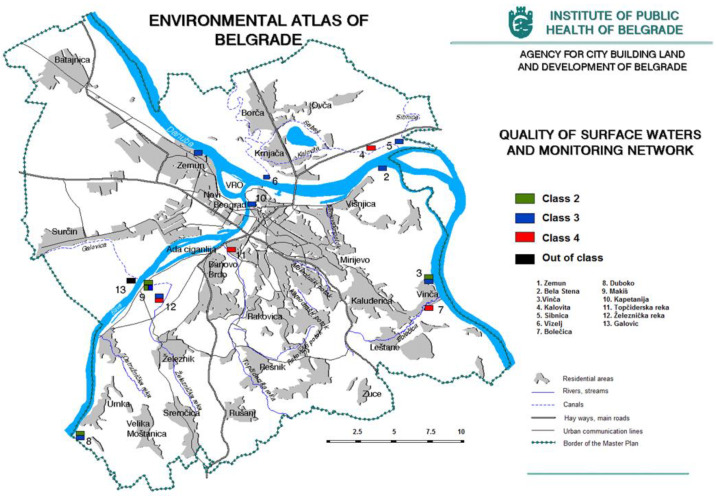
Quality of the surface water and monitoring network (https://www.zdravlje.org.rs/ekoatlas/volbe/39ev.gif, accessed on 23 August 2022).

The water quality of the Sava and Danube Rivers corresponds to the class III quality of watercourses (Figure 6). The main reason for the poor water quality is the pollution of both rivers upstream from Belgrade and also the discharge of industrial and communal wastewater from the territory of Belgrade directly into the Sava and Danube Rivers or through tributaries. The analysis of water samples in the tributaries shows high levels of pollution, and almost all samples are outside class II. The territory of Belgrade is also rich in streams that have been turned into collectors and channeled by urbanization, but in some parts of the city, they flow within a topographic surface. These watercourses are not monitored because they have been turned into wastewater collectors, so about their quality cannot be discussed [12].

**Figure 7:**
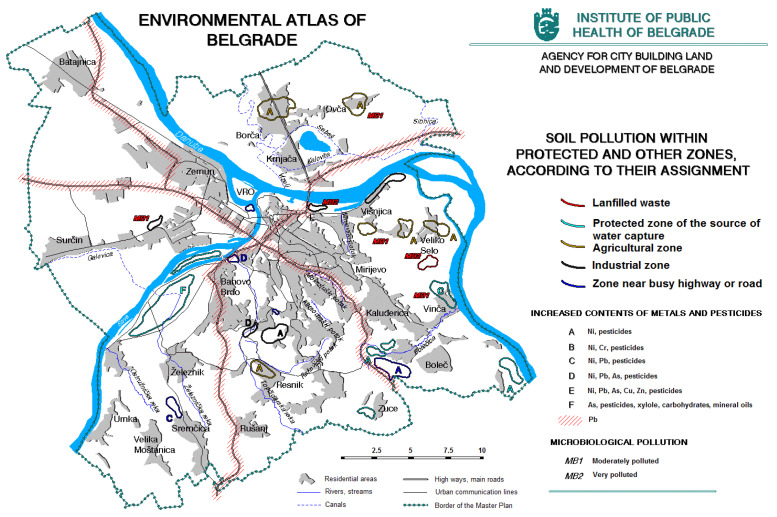
Soil pollution (https://www.zdravlje.org.rs/ekoatlas/volbe/45ev.gif, accessed on 23 August 2022).

In peri-urban and rural areas where agriculture is developed, the limiting factor of land use is the excessive use of pesticides and irrigation with polluted water from surface watercourses (Figure 7). In industrial zones, the landfills, industrial facilities, individual furnaces, and thermal energy complexes with their accompanying infrastructure have a great impact on the soil quality. Building land and fragmented green areas dominate the inner city [12].

Nature protection in the territory of the city of Belgrade is based on the preservation and sustainable use of natural resources and natural values [24,25,26]. In addition to the status of a protected area at the national level, ecologically important areas, ecological corridors, and ecological networks are of international importance for nature protection [24,27].

## 3. Results

### 3.1. Analysis of the Future UGI Development Practice of the City of Belgrade 

The balance of areas of planned forests and public green areas [12] is given in Table 1.

The realization of a solution for Belgrade’s green area system of 123 m^2^, including forests and green areas per capita, was to be realized by 2021.

It is planned to preserve and improve various types of forests and forest land with a total area of about 5744.41 ha (Table 1). Forests have a role in regulating air quality and temperature, as well as preserving the diversity of habitats and species. They are important in improving the aesthetic quality of an area or part of the city, as well as the overall picture of the landscape. The specificity of habitat conditions and the richness of forest types play a significant role in education. To improve the condition and general well-being of existing forests and forest land, it is necessary to conduct restorative or reconstructive work in high-diluted and/or degraded natural or artificially raised stands; carry out the conversion and/or reconstruction of coppice forests; preserve existing wetland habitats in the areas of forests and forest land; implement sanitary felling as a measure of forest care and protection; improve technical infrastructure (forest roads, fire-fighting railways and other facilities used for forest management); preserve and improve the existing forest nurseries and their affirmation through the tourism industry of Belgrade; and enter the forest land into the cadaster based on the existing bases of forest management (update the cadaster) [12]. Achieving the set goals of sustainable development of forests and forest land is planned to be achieved by raising new forests. New afforestation is planned on land extremely unfavorable for construction, areas of potential landslides, land of poorer quality (V, VI, and VII), in directions of dominant winds, along roads, in the area of underground water reservoirs, and in potential flood areas, etc. The following rules should be observed when establishing new forests: afforestation should be performed with tree species that correspond to natural potential vegetation, and by habitat conditions; priority should be given to indigenous species of woods; mixed and structurally diverse stands should be formed; the choice of species and method of planting should be adjusted to the basic function (protection from wind, landslides, water, biological reclamation of landfills, etc.); within the forest, gaps and meadows should be planned with a width equal to twice to five times the height of the surrounding trees—the following relationships should be strived for: open areas 15–20%, semi-open 10–15%, and closed areas (forests) 65–70% [12].

The long-term concept of urban development also implies the preservation of existing public green areas in a continuously built urban tissue and the planning of new ones to achieve publicly beneficial functions for the environment and society. They are planned as ecologically functional spaces to protect the environment, especially the regulation of microclimatic characteristics of urban areas, improving the image of the city, and achieving cultural, educational, and recreational functions. It is planned to preserve and improve various types of existing public green areas, with a total area of about 2209 ha (Table 1). Furthermore, it is planned to raise new public green areas of different types and their spatial and functional connection into a single system of green areas of the city, together with existing public green areas, forests, green areas for other public purposes, and green areas for other purposes.

The construction of new parks is planned for an area of about 454 ha (Table 1). Within the parks, wetland habitats can be formed, and contents in the park should be thematically concentrated and satisfy all age groups, taking into account the size of the green area, defined subtype of the park, the micro-location, its importance for the whole spatial context of the city, and the natural and cultural value of the space.

**Figure 8:**
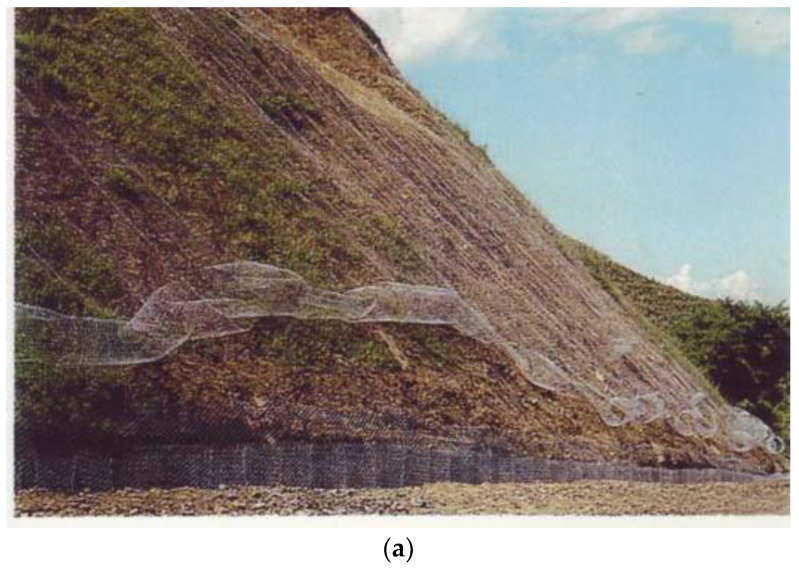

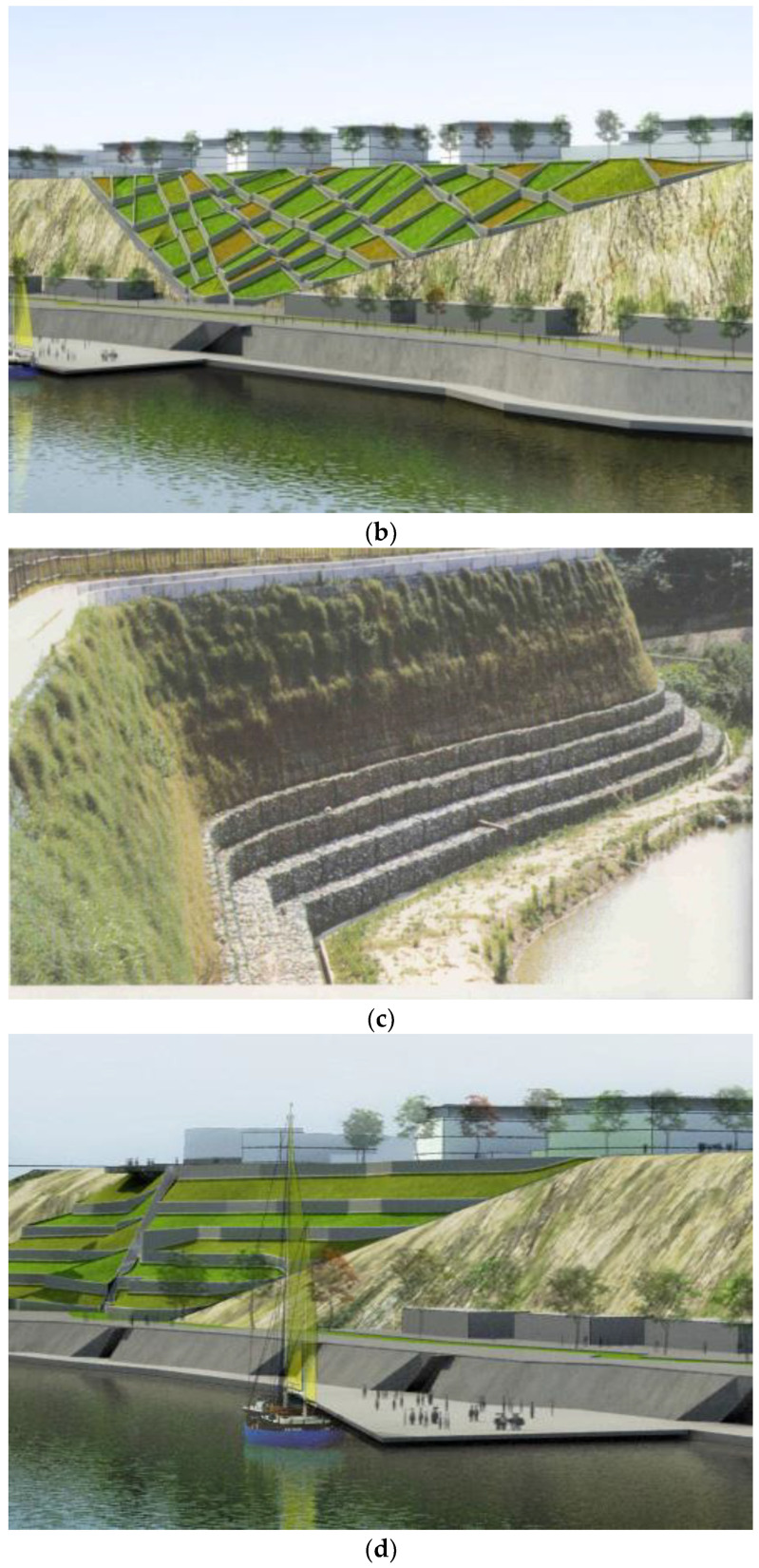
(**a**) Bio-shotcrete; (**b**) greenery in cassettes; (**c**) protective greenery, cascading decoration, and sub-wall; (**d**) cascading landscaping and bio-shotcrete (https://www.sllistbeograd.rs/pdf/2019/110-2019.pdf, accessed on 28 August 2022).

It is planned to build new squares on an area of about 25 ha, new green areas along the banks, in the inundation area of the Sava and Danube Rivers, and new protective green belts on areas of about 85 ha, 21 ha, and 1344 ha, respectively (Table 1). Green areas in the inundation areas of the Sava and the Danube Rivers are especially important for the conservation of biodiversity. They provide conditions for the movement and nutrition of fauna, and vegetation has a role in water purification, which is why their preservation is planned as part of the “Green link” system. New protective green belts have a specific function in protecting settlements and/or agricultural land from the negative effects of traffic (harmful exhaust gases), wind, and snow; residents from the negative effects of traffic (noise) and other activities that negatively affect the quality of the environment, and above all the health of the population; from natural disasters; from excessive visual barriers; from the harmful effects of erosion (water, aeolian); and biodiversity, etc. An example of one easily implemented NBS close to transport infrastructures is green belts formed by creeper plant species on simple bearing structures, which require limited care [14]. The protective green belt along the loess section has a role in strengthening the soil with the function of protecting the soil from erosion, but also in preserving and presenting the morphology and natural appearance of the section. The following geological–biotechnical measures are planned: protection of the section from erosion; prevention of leaching; mitigation of the steep slopes section; slopes must be additionally protected (with bio-shotcrete, fast-growing trees, etc.); establishing the continuity of green areas, regardless of the type of greenery; the preservation of existing vegetation, especially forests, but also the introduction of species more suitable for the basic function; and maintenance of the vegetation sprouted by natural succession (spontaneously) on vertical sections [12] (Figure 8a–d).

As the population pressure grows, the natural landscape is shattered, negatively impacting biodiversity. However, applied nature-based approaches change this tendency. For example, isolated natural reserves can be reconnected via green corridors [14]. To connect green spaces in Belgrade, it is planned to build new green corridors in an area of about 728 ha (Table 1). Some parts of the green corridor can be arranged as park areas, forests, protective green belts, or areas of natural vegetation (vegetation created by the process of natural succession). Within the green corridor, a wet habitat can be formed to improve the ecological function of the green infrastructure of the city [12].

It is planned to build new green areas for special purposes, including “collections of plants in the open” on an area of about 2 ha (Table 1). Wetlands have an essential role in hydrological cycles, supporting a richness of biodiversity, purifying contaminated water, and storing significant amounts of carbon. In Belgrade, new wetland habitats are allowed to be formed in areas where the habitat conditions are appropriate (high groundwater level, natural depressions, swampy areas, etc.). Wetlands can be formed within other public green areas, such as parks and green corridors, or can also be made into engineered systems, which are designed and constructed so as to utilize the natural functions of wetland vegetation, soils, and their microbial populations for the treatment of contaminants in surface waters, ground waters, or wastewaters [12]. It is increasingly required that developing systems use plants to purify wastewater (free water surface wetlands, horizontal subsurface flow wetlands, and vertical flow wetlands) before introducing it into the system or using it for other purposes (mostly irrigation) [14]. Within the circular-economy strategy, the EU commission encouraged the idea of water reuse [3]. Multi-purpose green infrastructure (constructed wetlands and parks) has equivalent or more acceptable performance than the gray infrastructure options at the same price.

**Figure 9:**
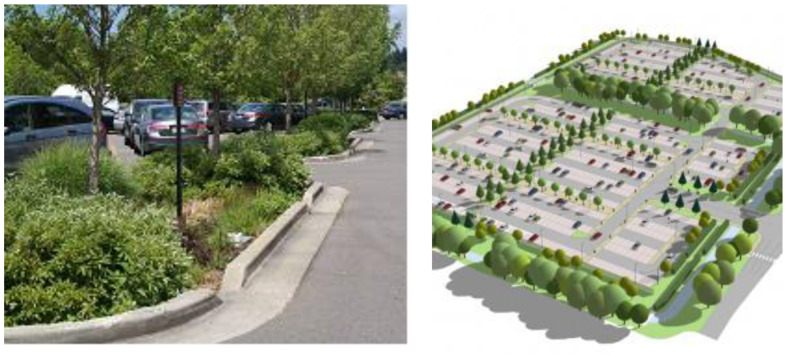
Grass gardens in the parking space (https://www.sllistbeograd.rs/pdf/2019/110-2019.pdf, accessed on 28 August 2022).

To establish the UGI of the city, it is planned to raise public green areas and forests within the open apartment blocks and other areas of public purpose (facilities and complexes of public services; traffic areas; facilities, complexes, and infrastructure corridors; and communal areas) (Table 1). When raising green areas in newly planned open apartment blocks, it is necessary to take into account the size of the green area and the spatial connection of the individual parts. Public green areas and forests within the areas of buildings and complexes of public services have a multifunctional role, where the primary role is their positive impact on improving the quality of the environment, as well as a positive role in preserving the city’s biodiversity, but also improving the physical and mental health of residents and users of facilities and spaces of a particular public service. Furthermore, these green areas deliver opportunities for the leisure and spontaneous recreation of residents from the immediate environment. When reconstructing the existing public green areas within the areas for facilities and complexes of public services, it is obligatory to respect the following rules: preserve the green area within the existing boundaries; preserve quality vegetation; rejuvenate existing vegetation; for landscaping, use indigenous types of vegetation that belong to natural potential vegetation, and which are adaptable to local environmental conditions; it is possible to use exotic specimens of that have been confirmed to adapt well to environmental conditions; the participation of deciduous species should be dominant in relation with other vegetation; use leafy decorative and floral forms of shrubby species and seasonal flowers; avoid invasive and allergenic species; the spatial functional organization and manner of arranging green areas should be in accordance with the needs of the primary purpose, spatial layout of buildings, their height and aesthetic design, the exposure and slope of the terrain, the depth and type of planting base, and the groundwater level; and provide a minimum % of the area under vegetation in direct contact with the ground. It is planned to raise extensive and intensive green areas on the roofs of buildings, as well as implement vertical landscaping on the facades of public buildings, and above-ground and underground garages of these complexes, all to improve microclimatic conditions and increase the energy efficiency of buildings. Green roofs help lower indoor temperatures by approximately 5 °C, improving biodiversity and mitigating the impact of the urban heat island [14]. Moreover, they are one of the most impressive solutions for compact and dense urban areas without green spaces [14]. In open parking spaces, it is necessary to envisage greening using semi-porous curtains with grass cover instead of impermeable curtains, planting tree seedlings, and/or forming grass gardens. Trees should be planted in the last third of the parking space, as follows: in case of administrative and oblique parking, plant one tree trunk in every two to three places (depending on the species); and when parking longitudinally, plant one tree in every two places. Grass gardens, in addition to standard landscaping, can be a SUDS, or rainwater management tool, designed to mimic natural drainage systems (Figure 9).

In the sanitary protection zones, it is permitted to plant grass, decorative greenery, and other plants that do not have deep roots, and which are exclusively natural, i.e., without the use of chemicals and fertilizers. Therefore, it is necessary to form a protective green (or forest) belt around the landfill perpendicular to the wind direction. Its structure should be impermeable, i.e., formed in the entire profile from the canopy of trees and shrubs, wherein preference should be given to deciduous trees [12].

The total area of city’s urban protected areas covers about 1.74% of the total area. Currently, 44 areas are protected, while 3 are in the process of being placed under protection. Of the natural monuments, as a spatial unit, 15 areas have been protected, and 3 are in the process of being placed under protection. Monuments of nature are categorized as monuments of the third category of local significance with the first or third degree of protection. The total number of landscapes with exceptional features is three, and they are characterized by very diverse flora and fauna. There are three levels of protection defining these protected areas and they represent extremely important areas identified through planning documentation. Urban protected areas close to urban population centers provide unique support for human health. They operate as carbon sinks while maintaining and improving the local biotope variety [14].

The long-term concept of urban development implies connecting urban tissue with the natural environment, natural and renewable resources, as well as planning the protection and development of existing forests. The system of green areas is planned in six spatial–functional units: I—core; II—inner ring; III—outer ring; IV—green connections; V—continuously constructed urban tissue; and VI—discontinuously built-up areas.

The harmony of the landscape of preserved natural, cultural, historical, and urban values represents the “Natural Core of Belgrade” with an area of about 9000 ha. The “Natural core of Belgrade” belongs to the central area of the ecological network of the Republic of Serbia (RS)—“The confluence of the rivers Sava and Danube”, which, in addition to national, has international significance. At the confluence of the rivers Sava and Danube, there exists a protected area called the Great War Island. The Great War Island is a dynamic ecosystem—a “living creature” that rivers continue to shape and to which complex natural processes can be traced. The preservation of exceptional natural values, as well as cultural–historical, and urban–architectural values of this area, their interaction, and integration, should be affirmed through the project of competent state administration bodies and institutions, which could propose this center of urban genesis (“genius loci”) of Belgrade for the UNESCO list of World Natural and Cultural Heritage. The “Natural Core of Belgrade” is at the same time the “core” of the planned system of green areas in Belgrade. Within this unit, the sustainable development of forests and green areas is planned, which means exemption from construction.

The “inner ring” of the green area system covers mostly the middle zone of the city, the area of the built city structure, which is dominated by the existing public green areas and forests. This area, with predominantly represents forests, city parks, public green areas within open apartment blocks, tree lines, and extremely rich vegetation within other public areas and areas with other purposes, has invaluable value for the citizens of Belgrade. Its value is reflected in better living conditions, an irreplaceable contribution to adaptation to current climate change, and the conservation of biodiversity, etc., which is why it should be fully preserved and improved. The preservation and improvement of the “inner ring” can be achieved through the planning of new forests, parks, squares, protective green belts, and tree lines, as well as by improving the percentage of green areas in direct contact with the ground within public areas and other purposes.

The “outer ring” of the green area system mainly includes the city’s peripheral zone, existing and newly planted forests, and forest land, as well as agricultural land. During further planning activities, it is necessary to develop this area in the direction of sustainability, i.e., to preserve and increase forest cover, as well as openness to nature close to the landscape with a network of green corridors along watercourses, hedges, and tree lines, etc.

The role of the “green connection” of the green area system is to connect the “core”, the “inner Ring“, and the “outer ring” into a complete system. Forests and green areas that build “green connections” are key elements of the city’s green infrastructure. Most of the parks and squares are extremely important as ecologically and aesthetically functional spaces in the urban tissue, prominent objects of the city’s landscape architecture that affirm the existing ambient, natural, and cultural values of Belgrade. Special emphasis should be placed on private yards in blocks of individual housing in the central zone of the city, which should be fully preserved, as well as the routes of the existing tree lines which are the main connections of the system of green areas.

The discontinuously built-up area includes open, predominantly agricultural areas and minimally built-up areas of the outer and edge zones. This whole, regardless of the large territorial distribution, includes smaller forests, forest remnants, shrubs, hedges, wetlands, marsh ecosystems, green areas within the backyards of suburban households, etc., whose connection plays a significant role in preserving biodiversity and nature [12].

### 3.2. Analysis of Planned Pathways for Mitigating the Effects of Climate Change in Belgrade 

The planned system of green areas in Belgrade is of key importance for mitigating the effects of climate change. However, in addition to the planned system of green areas, some solutions and measures can have a significant contribution to preserving biodiversity, nature, and natural processes, improving the quality of the environment, improving the energy efficiency of buildings, and mitigating the effects of climate change. Some of these solutions are as follows: increasing the ecological effect of the biotope in the plot (block) and reaching the set target “ecological index” (EI); forming energy plantations of fast-growing deciduous species (willow); forming systems for wastewater treatment and the revitalization of polluted waters by plants (constructed wetlands, reed bed system, floating treatment wetlands, floating islands); and planning of a sustainable drainage system (SUDS).

#### 3.2.1. Ecological Index (EI)

Continuously built urban tissue, especially the central parts of the city, is characterized by a large percentage of built and covered land, which causes extreme temperatures, reduced humidity, inadequate drainage of atmospheric water into the sewer system, reduce biodiversity, etc., or serious environmental problems. With the growing awareness of the problems we face, with the understanding of the functioning of natural systems in the urban environment and their importance, the model of sustainable development of cities has been improved by improving the functionality of existing ecosystems and forming new biotopes (habitats), while maintaining existing urban morphology and space. In many cities in Europe and America, in addition to standard urban parameters, this practice includes an ecological parameter, “Biotope Area Factor—BAF”, or “green factor” (GF) [28,29,30]. The importance of introducing this parameter is reflected in determining the relationship between ecologically valuable and other spaces at the plot level, optimized by the requirements of sustainable development of the central urban area. The application of this method ensures a certain amount of ecologically functional space on each building plot (block). In this way, the standardization of environmental quality management is achieved. Ecologically functional spaces on the plot, in addition to areas that are completely covered with vegetation and which, as ecosystems, naturally have the greatest positive effects, include covered semi-porous and porous surfaces, green roofs, facades of buildings, etc. Different weight factors have been defined, which qualitatively determine different types of ecologically functional spaces on the plot. For the urban parameters, on the territory of Belgrade, it is necessary to introduce an EI after the minimum target EI is defined for urban forms of different purposes and types of construction. Having in mind the determination of the city to base its development on the strategy of sustainability, which results in the needs and obligations for the implementation of necessary measures for adaptation of the city to climate change, the EI can constitute a special contribution to sustainable development.

#### 3.2.2. Energy Plantations of Fast-Growing Deciduous Species

For the long-term supply of clean energy to the territory of Belgrade, forming energy plantations of fast-growing deciduous species, i.e., willows [31] is proposed. Energy willow (*Salix viminalis*), with its varieties adapted to local climatic conditions and soil conditions, is the correct answer to questions related to achieving high yields of wood mass per hectare in a short time [31]. When burnt, it releases a negligible number of pollutants. The amount of CO_2_ and CO released by its combustion is almost equal to zero. In addition, to the above mentioned, it has a positive impact on the conservation and protection of ecosystems, their restoration, the reusing of nutrients, and soil conservation through protection against wind and water erosion. Integrating energy plantations into the system of green areas in Belgrade would achieve multiple benefits, especially in improving the quality of the environment.

#### 3.2.3. Systems for Wastewater Treatment and Revitalization of Polluted Waters by Plants

The effective disposal of wastewater means its release into the environment without adverse effects on human health, natural resources and biodiversity, the visual quality of the landscape, etc. In Belgrade, the application of wastewater treatment through aquatic plants as a specially constructed system is proposed. A suitable example of such a system in nature is wetlands [32,33]. The constructed wetland systems are based on the symbiotic relationships that prevail in natural wetland ecosystems, in the interconnection of substrate and water. The aim is to exploit complex ecological links in the construction of a “food chain” that allows waste products to be decomposed and assimilated into plant or animal biomass. In combination with this system, ponds can be used. Some of the technical arguments for using ponds in conjunction with wetlands are that wetlands take years to develop the complex biodiversity needed to achieve effects in stopping heavy loads, and they are particularly vulnerable in the first six months after establishment. The biggest gain of using the pond system is its rapid response to high loads [34]. Because algae and bacteria have a short breeding season, they can increase the level of populations according to the received load [34]. In addition to wastewater treatment, this system can also be used for the treatment of rainwater (atmospheric water), water from industrial plants, and for water treatment in watercourses [33].

#### 3.2.4. Sustainable Urban-Drainage System (SUDS)

**Figure 10:**
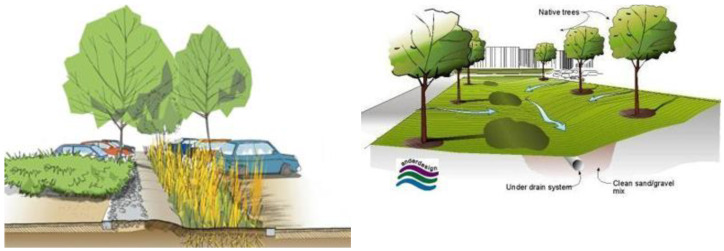
Plan for the sustainable drainage system in Belgrade (https://www.sllistbeograd.rs/pdf/2019/110-2019.pdf, accessed on 28 August 2022).

In the natural water cycle, most atmospheric water sinks (infiltrates) into the soil, and only a small part is surface-directed into the sewer [35]. In this way, groundwater is naturally regenerated and the natural balance is maintained [36]. The natural soil layer, thanks to its ability to purify water, performs the function of groundwater protection during the long-term and effective infiltration of rainwater into green areas [37]. With increased construction, more and more urban spaces in Belgrade are turned into impermeable surfaces, and atmospheric water must be drained through canals, which leads to negative consequences, which include the following: rapid and large inflow of water into the sewer (which must therefore be designed for such conditions); the occurrence of high water levels; the necessity of additional construction of watercourses; the reduction of groundwater levels; the deterioration of the microclimate; and the devastation of natural landscapes. A SUD_S_ is a rainwater management tool designed to mimic natural drainage systems. There exist various planned techniques which make the efficient management of atmospheric water drainages possible, such as wetlands, water pools, rain gardens, planting pits and gardens, green trenches, semi-porous curtains, bioretention, green roofs, canals, and many other measures, which all have the goal to infiltrate atmospheric water into watercourses or land (Figure 10).

### 3.3. Analysis of the Success of NBS Implementation 

In urban areas, the successful implementation of NBS depends on the following five key steps: (I) determination of city characteristics and specific problems; (II) assessments of positive and negative impacts of the NBS; (III) ensuring an adequate financing stream; (IV) developing policies and incentives; (V) dissemination of information. NBS is classified into three different types based on the degree of intervention/level and engineering type in the different applied solutions [12]. After reviewing the typology, NBS categories are classified into Type 1—more acceptable usage of protected/natural ecosystems; Type 2—NBS for sustainability and multifunctionality of managed ecosystems; and Type 3—creation and managing of new ecosystems [14]. Based on the degree of the modification of nature and the landscape in the examined case study area, a modern model of integral and integrative nature protection will be applied. The strategic commitment to the protection of nature in Belgrade implies efficient and consistent implementation of legal provisions in the process of city planning, in the field of nature protection, and environmental protection, as well as the affirmation of nature protection, primarily by raising the awareness and education of citizens. Following the degree of intervention/level and engineering type in Belgrade, it is intended as Type 3—design and management of new ecosystems in the categories of intensive urban green space management; urban planning strategies; urban water management; ecological restoration of degraded terrestrial ecosystems; and restoration and creation of semi-natural water bodies and hydrographic networks. 

Every single NBS type implies favorable environmental influences. Nevertheless, if the planning is not supported by scientific evidence, there are potential risks. The true capacity of NBS in environmental improvement is highly vulnerable. For example, including trees in cities to obtain advantages such as carbon sequestration and reducing the heat island impact may also increase fire risks [38], allergic reactions [39], and emissions of biogenic volatile organic compounds [40]. Concerning vegetation, using the wrong species may not deliver a cooling effect but deepen the heat impact [41,42]. Unwanted plant and invasive species pests or diseases may be spread to new areas with long-distance transportation of plant materials and substrate. Selecting adsorbing tree species and absorbing the maximum amount of air pollutants is essential in reducing air pollution. The tree placement also ought to be evaluated to ensure that pollution is not caught and contained in high-traffic areas [43]. Furthermore, NBS provides ample opportunity for biodiversity protection. The type and number of species used in NBS will define the result. Unprofessional implementations may result in undesirable effects. NBS should be established on indigenous species in a broad geographic sense, considering plant origins. To save species affected by climate change, NBS should benefit from assisted migration by delivering unique and appropriate habitats to address climate change and the endangerment of plant and animal species.

**Figure 11:**
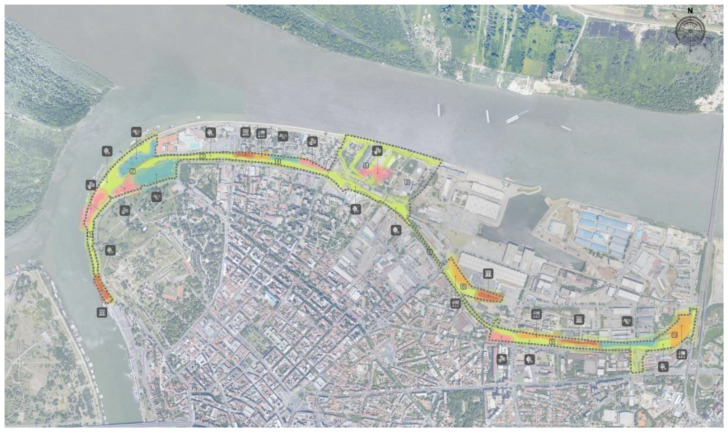
Plan to build the “Linear Park” (http://bellab.rs/wp-content/uploads/2021/03/CLEVER-Cities-_-TNOC.pdf, accessed on 30 August 2022).

To ensure sustainability and quality, all the infrastructure services demand an adequate financing stream. Public budgets are often obtained from three primary sources: tariffs, taxes, and transfers [44]. Some projects finance and support the integration of green components in the restoration of public squares and streets, the creation of green corridors between greened areas, and contribute to the natural restoration. Across Europe, most NBS projects are focused on ecosystems, emphasizing their functionality, integrity, and connectivity. NBS projects focused on community gardens and urban parks primarily contribute to human well-being, while NBS projects focused on streams, rivers, and estuaries are mainly focused on ecosystem restoration. One of those projects is Horizon 2020—CLEVER Cities. The aim of the CLEVER Cities project is to conduct a unique kind of nature-based urban modification for sustainable and socially inclusive cities across Europe, South America, and China. Belgrade, Larissa, Madrid, Malmö, Sfântu Gheorghe, and Quito share and learn with London, Hamburg, and Milan regarding how to adjust nature-based interventions for the demands of urban areas worldwide. Within this project in Belgrade is a planned Linear Park (Figure 11). 

The Linear Park will be constructed on a derelict railway track previously used to transport hazardous waste, bordering the historic Belgrade Fortress and crossing residential and industrial areas. Polluted land will undergo a soil-remediation procedure and will be converted into green spaces where residents can escape hectic urban life and bond with nature, enjoy art, stroll through parks, and discover local history. This area will be a corridor to the central zone of the city, and it is inspired by the linear parks in New York and Moscow. The trees and natural features of the park will act as carbon sinks and will help cool down Belgrade amidst increasing temperatures caused by climate change. They will also control flooding and populate green areas and gardens with playgrounds.

**Figure 12:**
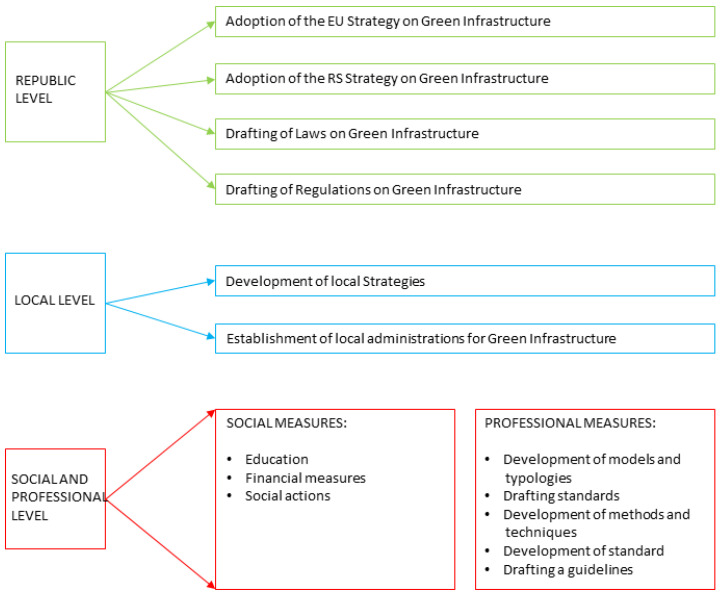
Legal framework RS and green infrastructure disadvantages and potential (http://bellab.rs/wp-content/uploads/2021/03/CLEVER-Cities-_-TNOC.pdf, accessed on 30 August 2022).

It is essential to develop policies and incentives that help upgrade the usage of NBS in urban infrastructure development. The first step is the harmonization of legislation at international, national, regional, and local scales for facilitating NBS uptake and sustainable development in general. The second step is the integration of NBS into the existing legal and institutional context and implementation at national, regional, and local levels. All strategic documents can efficiently support nature-based solution propagation. Green infrastructure as a concept has yet to be established in the legislation of the Republic of Serbia (RS) and there is no systematic approach to this issue. Several laws and initiatives are or should be in contact with UGI, but in most laws access to green infrastructure is partial or completely absent, for example, in the Law on Environmental Protection (“Official Gazette of RS”, no. 135/2004, 36/2009, 36/2009, 72/2009, and 43/2011), Law on Nature Protection (“Official Gazette of RS”, no. 36/2009, 88/2010, 91/2010, 14/2016, and 95/2018), and Law on Forests (“Official Gazette of RS”, no. 30/2010, 93/2012, 89/2015, and 95/2018), which do not recognize forests in the context of UGI. In public policy documents at the national and local levels, the notion of UGI has begun to emerge in recent years. At the national level, the principles of the NBS are recognized in the Sustainable Urban Development Strategy of the Republic of Serbia until 2030, and at the local level in the General Regulation Plan of Belgrade Green Areas (2019), Action Plan for Climate Change Adaptation for City of Belgrade (2015), and Environmental Protection Program (2015). To address multiple climate and urbanization-related challenges taking into account positive environmental impacts in the future, the RS has the task of adopting the EU Strategy on Green Infrastructure, the RS Strategy on Green Infrastructure, and drafting laws and Regulations on Green Infrastructure (Figure 12).

The fundamental performers for sustainable land use and planning, including the NBS use, are local, regional, and national authorities, the media, and the public. Education at all levels about climate change, NBS, and sustainability can provide learners with the necessary skills [45,46,47]. Education for green infrastructure focuses on improving public education concerning the advantages of green infrastructure, which could raise management, public approval, and stewardship of current and upcoming green infrastructure projects. In addition, it refers to the vast learning possibilities delivered by city infrastructure projects, where ecosystem services are entangled with human development, teaching fundaments about systems sustainability, thinking, and resilience, and to the rich opportunities for place-based education in cities. 

## 4. Conclusions

Based on a case study from an urban environment that deals with the local future planned process of regeneration of the city with the function of changing the physical organization, improving biodiversity, ecosystem health and resilience, and mitigating climate change, Belgrade has planned several green infrastructure designs through NBS to address multiple climate and urbanization–related challenges taking into account positive environmental impacts. The proposed measures for increasing UGI designs through NBS for sustainable urban development in the territory of Belgrade correspond to Type 3 of NBS categories—design and management of new ecosystems. Planning solutions will be based on the available international theoretical and experiential knowledge about the importance of forests and green areas in urban areas. In particular, the proposed solutions cover all purposes of space (public and other) by prescribing minimum measures for implementing and arranging green areas and forests within them, thus integrating nature protection and environmental protection measures into the built urban tissue. 

While NBS is frequently more cost-effective than traditional gray infrastructure options, the hindrances and bottlenecks for implementation are complicated and are associated with securing investment, management, education, and working collaboration, etc. The current decision-making mechanisms recognize the importance of the NBS to embark on the path of shaping cities; however, UGI as a concept has yet to be established in the legislation of the RS. In the future, it is important to create policies and incentives on national, regional, and local scales that assist in boosting the use of GI designs through NBS for sustainable urban development. Furthermore, it is imperative that urban planners across the region understand the importance of UGI design through NBS for enhancing resilience in built-up environments.

## Figures and Tables

**Table 1:** The balance of areas of planned forests and public green areas [12].

Types of Existing Green Areas	Existing		Planned Change of Purpose	Planning		Total	
	ha	%	ha	ha	%	ha	%
Forest and forest land	7444.50	9.55	−336.13	5739.08	7.36	12,847.45	16.48
Forest and forest land	7040.60	9.03	−336.13	5744.41	7.37	12,448.88	15.97
Wetlands on forest land	403.90	0.52	0.00	−5.33	−0.01	398.57	0.51
Urban green area	2208.90	2.83	0.00	2669.91	3.42	4878.81	6.26
Parks	258.90	0.33	0.00	453.29	0.58	712.19	0.91
Squares	15.60	0.02	0.00	26.72	0.03	42.32	0.05
Green areas along the banks of the Sava and Danube Rivers	158.30	0.20	0.00	85.55	0.11	243.85	0.31
Green areas in the inundation area of the Sava and Danube Rivers	68.40	0.09	0.00	20.75	0.03	89.15	0.11
Green areas: open apartment blocks	1121.10	1.44	0.00	15.52	0.02	1136.62	1.46
Protective green belts	394.80	0.51	0.00	1344.08	1.72	1738.88	2.23
Green corridors	8.70	0.01	0.00	722.77	0.93	731.47	0.94
Green areas of special purpose	9.90	0.01	0.00	1.98	0.00	11.88	0.02
Wetlands	170.50	0.22	0.00	−0.78	0.00	169.72	0.22
Nurseries	2.70	0.00	0.00	0.02	0.00	2.72	0.00
Total	9653.40	12.38	−336.13	8408.99	10.79	17,726.26	22.74

## Data Availability

The data will be made on reasonable request.

## References

[1] Fink S.H. (2016). Human-nature for climate action: Nature-based solutions for urban sustainability. Sustainability.

[2] Cohen-Shacham E., Walters G., Janzen C., Maginnis S. (2016). Nature-Based Solutions to Address Societal Challenges.

[3] European Commission (2015). Towards an EU Research and Innovation Policy Agenda for Nature-Based Solutions & Re-naturing Cities (Final Report of the Horizon 2020 Expert Group on Nature-Based Solutions and Re-naturing Cities).

[4] Eggermont H., Balian E., Azevedo J.M.N., Beumer V., Brodin T., Claudet J., Fady B., Grube M., Keune H., Lamarque K. (2015). Nature-based solutions: New influence for environmental management and research in Europe. GAIA.

[5] Egorov A.I., Mudu P., Braubach M., Martuzz M. (2016). Urban Green Spaces and Health: A Review of Evidence.

[6] Cole B.L., McPhearson T., Herzog P.C., Russ A., Russ. A., Krasny E.M. (2017). Green infrastructure. Urban Environmental Education Review.

[7] Oijstaeijen V.W., Passel V.S., Cools J. (2020). Urban green infrastructure: A review on valuation toolkits from an urban planning perspective. J. Environ. Manag..

[8] Carrus G., Scopelliti M., Lafortezza R., Colangelo G., Ferrini F., Salbitano F., Agrimi M., Portoghesi L., Semenzato P., Sanesi G. (2015). Go greener, feel better? The positive effects of biodiversity on the well-being of individuals visiting urban and peri-urban green areas. Landsc. Urban Plan..

[9] Townsend M., Thrush S.F., Lohrer A.M., Hewitt J.E., Lundquist C.J., Carbines M., Felsinge M. (2014). Overcoming the challenges of data scarcity in mapping marine ecosystem service potential. Ecosyst. Serv..

[10] Faehnle M., Tyrväinen L. (2013). A framework for evaluating and designing collaborative planning. Land Use Policy.

[11] Mesimäki M., Hauru K., Kotze D.J., Lehvävirta S. (2017). Neo-spaces for urban livability? Urbanites’ versatile mental images of green roofs in the Helsinki metropolitan area Finland. Land Use Policy.

[12] (2019). General Regulation Plan for Green Areas System of Belgrade.

[13] Yilmaz S., Mumcu S., Efe R., Curebal I., Gad A., Toth B. (2016). Urban Green Areas and Design Principles. Environmental Sustainability and Landscape Management.

[14] Somarakis G., Stagakis S., Chrysoulakis N. Thinknature Nature-Based Solutions Handbook.

[15] Gill S.E., Handley J.F., Ennos A.R., Pauleit S. (2007). Adapting cities for climate change: The role of the green infrastructure. Built Environ..

[16] Banđur V., Potkonjak N.M. (1999). Metodologija Pedagogije.

[17] (2015). Climate Change Adaptation Action Plan and Vulnerability Assessment.

[18] (2015). Environmental Protection Program of the City of Belgrade.

[19] (2018). City of Belgrade Development Strategy: Goals, Concept and Strategic Priorities of Sustainable Development.

[20] Aranđelović B., Vukmirović M., Samardzić N. (2017). Belgrade: Imaging the future and creating a European metropolis. Cities.

[21] (2016). Master Plan of Belgrade.

[22] (2007). Environment in the City of Belgrade.

[23] (2016). Law on Waters.

[24] Law on Nature Protection. https://www.pregovarackagrupa27.gov.rs/wp-content/uploads/2021/06/LAW-ON-NATURE-PROTECTION-2016.pdf..

[25] (2004). Law on Environmental Protection.

[26] (2013). Regulation on the Ecological Network.

[27] (2010). Decree on the Ecological Network.

[28] Kruuse A. (2011). The green space factor and the green points system.

[29] Farrugia S., Hudson M.D., McCulloch L. (2013). An evaluation of flood control and urban cooling ecosystem services delivered by urban green infrastructure. Int. J. Biodivers. Sci. Ecosyst. Serv. Manag..

[30] Huang Z., Yu H., Peng Z., Zhao M. (2015). Methods and tools for community energy planning: A review. Renew. Sustain. Energy Rev..

[31] Aravanopoulos F.A. (2010). Breeding of fast-growing forest tree species for biomass production in Greece. Biomass Bioenergy.

[32] Brix H., Arias C.A., Johansen N.H., Vymazal J. (2003). Experiments in a two-stage constructed wetland system: Nitrification capacity and effects of recycling on nitrogen removal. Wetlands—Nutrients, Metals and Mass Cycling.

[33] Vymazal J. (2005). Constructed wetlands with horizontal sub-surface flow and hybrid systems for wastewater treatment. Ecol. Eng..

[34] Ho L.T., Goethals P. (2020). Municipal wastewater treatment with pond technology: Historical review and future outlook. Ecol. Eng..

[35] Momparler Perales S., Andrés Doménech I., Hernández Crespo C., Vallés-Morán F.J., Martín Monerris M., Escuder Bueno I., Andreu Álvarez J. (2017). The role of monitoring sustainable drainage systems for promoting transition towards regenerative urban built environments: A case study in the Valencian region, Spain. J. Clean Prod..

[36] Norton B.A., Coutts A.M., Livesley S.J., Harris R.J., Hunter A.M., Williams N.S.G. (2015). Planning for cooler cities: A framework to prioritise green infrastructure to mitigate high temperatures in urban landscapes. Landsc. Urban Plan..

[37] Jia H., Wang Z., Zhen X., Clar M., Yu S.L. (2017). China’s sponge city construction: A discussion on technical approaches. Front. Environ. Sci. Eng..

[38] Lehvävirta S. (2007). Non-anthropogenic dynamic factors and regeneration of (hemi)boreal urban woodlands: Synthesising urban and rural ecological knowledge. Urban For Urban Green..

[39] Cariñanos P., Adinolfi C., Díaz de la Guardia C., De Linares C., Casares-Porcel M. (2016). Characterization of allergen emission sources in urban áreas. J. Environ. Qual..

[40] Livesley S.J., McPherson E.G., Calfapietra C. (2016). The urban forest and ecosystem services: Impacts on urban water, heat, and pollution cycles at the tree, street, and city scale. J. Environ. Qual..

[41] Solcerova A., van de Ven F., Wang M., Rijsdijk M., van de Giesen N. (2017). Do green roofs cool the air?. Build. Environ..

[42] Peng J., Dan Y., Qiao R., Liu Y., Dong J., Wu J. (2021). How to quantify the cooling effect of urban parks? Linking maximum and accumulation perspectives. Remote Sens. Environ..

[43] Ghasemian M., Amini S., Princevac M. (2017). The influence of roadside solid and vegetation barriers on near-road air quality. Atmos. Environ..

[44] Browder G., Ozment S., Rehberger Bescos I., Gartner T., Lange G.M. (2019). Integrating Green and Gray: Creating Next Generation Infrastructure.

[45] Yin S., Kasraian D., van Wesemael P. (2022). Children and urban green infrastructure in the digital age: A systematic literature review. Int. J. Environ. Res. Public Health.

[46] Klein Y., Lindfors P., Osika W., Magnusson Hanson L.L., Stenfors C.U.D. (2022). Residential greenspace is associated with lower levels of depressive and burnout symptoms, and higher levels of life satisfaction: A nationwide population-based study in Sweden. Int. J. Environ. Res. Public Health.

[47] Xu H., Fu F., Miao M. (2022). What is the effect of cultural greenway projects in high-density urban municipalities? assessing the public living desire near the cultural greenway in central Beijing. Int. J. Environ. Res. Public Health.

